# First-time mothers’ experiences of foetal reduction in pregnancy following assisted reproductive technology treatment in Taiwan: a qualitative study

**DOI:** 10.1186/s41043-021-00270-x

**Published:** 2021-11-02

**Authors:** Mei-Zen Huang, Yi-Chin Sun, Meei-Ling Gau, Shuby Puthussery, Chien-Huei Kao

**Affiliations:** 1 Department of Nursing, National Tainan Junior College of Nursing, 78, Sec.2 Minzu Rd., Tainan City, Taiwan; 2 Dr. Hwang Reproductive Fertility Center, 11F., No.100, Sec.2, Nanjing E. Rd., Zhongshan Dist., Taipei City, Taiwan; 3 Department of Midwifery and Women Health Care, National Taipei University of Nursing and Health Sciences, 365, Ming-Te Road, Peitou, Taipei, Taiwan; 4 Institute for Health Research and School of Society, Community and Health, University of Bedfordshire, Park Square Rm 206, Luton, LU1 3JU UK

**Keywords:** Maternal health, Foetal reduction, Assisted reproductive technology, Taiwan

## Abstract

**Background:**

Foetal reduction—removal of one or more foetuses to reduce the number of foetuses in multiple conceptions—is a procedure used for improving pregnancy outcomes following assisted reproductive technology (ART) treatment. While there is a recognition of the importance of understanding the experiences of women who undergo foetal reduction to offer appropriate help and support, studies that provide relevant insights are sparse. Our aim was to gain an in-depth understanding about first-time mothers’ experiences of foetal reduction following ART treatment in Taiwan.

**Methods:**

We adopted a qualitative design based on a phenomenological approach for this study. In-depth semi-structured interviews were conducted with seven first-time mothers who underwent foetal reduction following ART treatment at a fertility centre in Taipei, Taiwan. All interviews were recorded, transcribed and analysed using the Colaizzi strategy.

**Results:**

The views and experiences relating to foetal reduction reflected five key themes: hesitation, ambivalence and distress; the guilt of knowingly terminating a life; rituals and ceremonies to ease the sense of guilt; persuading oneself to consider the ‘big picture’; and wishing for a reunion in next pregnancy**.** Mothers often regretted that they took clinical advice to implant multiple embryos and then having had to resort to foetal reduction. There was a sense of hesitation, ambivalence and distress reflected in the views from all participants. They believed that they ended the fetuses’ lives knowingly and expressed strong feelings of guilt. Mothers often tried to persuade themselves to look at foetal reduction within the ‘big picture’ of the overall pregnancy outcome. Losing their unborn babies was as an unforgettable incident for most mothers, and they wished for a reunion with the lost baby in the next pregnancy.

**Conclusion:**

Findings indicate the need for ART providers to undertake a more sensitive approach that involves detailed discussions with women and their families to tailor the embryo transfer processes to suit individual needs. Women who undergo foetal reduction should be provided with tailored interventions towards enhancing their coping strategies before and after foetal reduction taking into account the cultural and religious context.

## Introduction

Assisted reproductive technology (ART) is increasingly being used as an acceptable method to enable women with untreatable infertility to conceive healthy babies. More than seven million babies were born worldwide within the last four decades as a result of successful ART treatment [1]. ART treatment is often linked to the likelihood of multiple conceptions [2] that carries significant implications for the health of the mother and the foetus and a higher risk of maternal and perinatal morbidity [2]. Researchers have linked twin conceptions to various complications for the mother and child [3]. Studies examining birth outcomes of higher-order multiple gestations have shown that such pregnancies carry greater risk of morbidity and mortality [2, 4] and adverse conditions and outcomes such as gestational hypertension, pre-eclampsia, thrombophlebitis, preterm birth, intrauterine growth retardation, congenital anomalies and stillbirth [5–7].

Foetal reduction, defined as the removal of one or more foetuses to reduce their number to one or two, is a method used for improving pregnancy outcomes following ART treatment [8]. Foetal reduction is used to reduce pregnancy loss, pregnancy related-complications and morbidity and mortality of the new born, and to improve birth weight and gestational age [9]. Foetal reduction procedure is usually carried out using three techniques: transabdominal foetal reduction, transvaginal foetal reduction, transcervical aspiration or suction [10, 11] with the latter two procedures employed more often [12].

Studies have investigated pregnancy outcomes following foetal reduction in twin and triplet pregnancies [13–19]. A meta-analysis of non-randomised studies suggested that foetal reduction to twins against expectant management of a triplet pregnancy is linked to lower rates of pregnancy complications and loss, preterm birth, caesarean section, low birth weight and neonatal mortality [13]. Women who underwent foetal reduction to reduce the number of foetuses to two had similar pregnancy outcomes as women who had conceived twin pregnancies naturally or through ART [20]. A study involving 255 women with triplet pregnancies, amongst which 185 underwent foetal reduction and reduced the number of foetuses to two, reported lower rates of preterm birth in the foetal reduction group compared to those who did not undergo foetal reduction (11.7% and 40.58% delivery at weeks 32 and 35 in the foetal reduction group vs. 36.76% and 83.82% at weeks 32 and 35 in the non-reduced group). Furthermore, there was a considerably higher percentage of babies born with low birth weight in the non-reduced group [21]. However, randomised controlled trials (RCTs) that identify the benefits and adverse outcomes of foetal reduction for women who have multiple gestations are relatively sparse.

Some researchers have explored women’s decision making and their emotional and psychological responses to foetal reduction [22–28]. Women tend to become highly anxious and stressful during the period when they consider foetal reduction to the time when they undergo the procedure [22]. Typically, this period begins when a couple discover that the woman is carrying more than one foetus (around 8 weeks after conception), and it usually ends around the twelfth week of pregnancy. This period is seen to be highly stressful and is often reported to be a turning point for couples as they are required to consider options for managing the multifoetal pregnancy [22, 29]. Garel et al. [23] found that amongst 18 pregnant women contemplating on foetal reduction, 12 women experienced excessive levels of stress and pain, and they wished to escape the situation. While women may continue to experience guilt and sadness about the loss of the foetuses [25, 30], the likelihood of miscarriage following the procedure is also a source for anxiety [31].

In Taiwan, the total fertility rate in 2019 was 1.05 children per woman [32]. Large majority (96.09%) of the births were single. Among the rest, twins and ttriplets accounted for 3.87% and 0.04%, respectively [33]. Approximately 20% of infertile women undergo ART treatment and approximately 4.33% of babies are conceived through ART treatment [34]. Although there is no official statistics on the rate of foetal reduction, it is an accepted practice for women undergoing ART treatment to undertake foetal reduction in Taiwan. While there is a recognition about the importance of understanding the experiences and feelings of women who undergo foetal reduction in order to offer appropriate help and support, studies that offer relevant insights are sparse in this area. A qualitative study exploring the experiences of six women who had undergone foetal reduction in Taiwan found that women experienced difficulties in accepting the unanticipated multi-foetal gestation, feared the risks of multi-foetal gestation and foetal reduction procedures, and experienced feelings of guilt with extreme levels of psychological distress [28]. Another study [35] explored the experiences of 10 participants who received chorionic villus sampling (CVS) to determine chromosomal or genetic disorders in the foetus before undergoing foetal reduction. The authors categorised their lived experience into seven themes covering different stages: (a) pre-foetal reduction: feeling threatened by the confirmed diagnosis of multifetal pregnancy, facing guilt and conflict of undergoing foetal reduction; (b) undergoing foetal reduction: getting confused due to family's concern about foetal reduction, losing a sense of body boundary intactness, and worrying about the safety of the remaining foetuses; (c) post-foetal reduction: grieving for losing foetus, returning to the course of normal pregnancy. The authors concluded that foetal reduction impacted the physical and psychological well-being of multi-feotal pregnant women [35]. While these studies offered some relevant insights, there is a need for more evidence to gain an in-depth understanding of women’s experiences of foetal reduction to inform policy, practice and future research in the area.

The aim of the current study was to gain an in-depth understanding about first-time mothers’ experiences of foetal reduction in pregnancy following ART treatment in Taiwan.

## Methods

We conducted in-depth semi-structured interviews as part of a larger qualitative study based on a phenomenological approach on first-time mothers’ experiences of pregnancy and transition to parenthood following successful ART treatment in Taiwan. We have previously reported the findings from the larger study on the overall experiences of pregnancy and birth [36]. The data presented in this article are derived from semi-structured interviews with seven first-time mothers who underwent foetal reduction following ART treatment at a fertility centre in Taipei, Taiwan. All the women gave live birth to their first baby following ART treatment and foetal reduction; they did not have a history of medical complications such as high blood pressure, diabetes and heart disease before they got pregnant; and they could understand, speak, read and write Mandarin.

After gaining relevant ethics approval from the institutional review board of Shin Kong Wu Ho-Su Memorial Hospital Taiwan, the principal investigator (MZH) approached potential participants at the fertility clinic at the hospital with an information leaflet. Those who expressed an interest to taking part after the initial discussion were given detailed information about the study and what participation involved. The researcher obtained written consent from participants before enrolling them in the study.

The characteristics of the participants are presented in Table 1. Their ages ranged from 31 to 39 years. All were married. Except one, all women underwent two or more cycles of invitro fertilisation (IVF) treatment with three or more embryos transferred, resulting in the conception of three or more live foetuses. Five women had one foetus reduced, and two women had two foetuses reduced. Following foetal reduction, all the women gave birth to twin babies. The gestational age at birth varied from 31 to 38 weeks.

**Table 1:** Participant characteristics

Participants	A	B	C	D	E	F	G
Age	31	33	35	39	35	36	36
Education	Bachelor’s degree	Bachelor’s degree	Master’s degree	Bachelor’s degree	Bachelor’s degree	Bachelor’s degree	Bachelor's degree
Religion	Taoism	Buddhism	Folklore	Folklore	Folklore	Buddhism	Buddhism
Cause of infertility	Female/tubal	Male/tubal	Male	Male	Ovulation	Ovulation	Male
Gravida (G)/parity (P)/spontaneous abortion (SA)/artificial abortion (AA)	G1P1	G2P1SA1	G1P1	G1P1	G5P2SA3	G2P1SA1	G5P1AA4
Number of IVF cycles	2	1	2	2	3	2	2
Duration of embryo transfer (day 2 through day 5)	D3	D3	D2	D3	D5	D2	D3
Number of transferred embryos	4	4	4	4	3	4	4
Number of live feotuses	3	3	4	3	3	4	3
Number of feotuses reduced	1	1	2	1	1	2	1
Number of live births	2	2	2	2	2	2	2
Gestational age at birth	33	36	31	38	37	36	37

The in-depth interviews took place 8–18 weeks after the women gave birth as part of the main study. The first author (MZH), who has seven years of nursing experience in the delivery room, postpartum ward and paediatric care unit of the hospital, conducted all of the interviews. The researcher conducted the interviews at the participants’ homes at a mutually convenient prearranged time, and there was no one else present apart from the researcher and the participant during the interview. The researcher used a flexible topic guide to guide the interviews. The women were first asked in general about their pregnancy and childbirth and then about the experiences of and feelings towards the ART treatment process and their subsequent motherhood. The original topic guide for the main study included questions on experiences during treatment, decisions and experiences about foetal reduction, reactions from the extended family, how they coped with family chores and employment, views about baby’s health and well-being, and expectations about additional support required from health care professionals before and during pregnancy, at childbirth, and after the delivery [36]. The information presented in this paper are mainly derived from questions concerning decisions and experiences relating to foetal reduction.

The process of the interview is described in detail elsewhere [36]. The interviews lasted between 1–2 h, guided by the woman’s desire to talk. The researcher gave the interviewees time to think about and reflect upon their experiences before the start of the interviews and assured the participants that anything they said would be valued, respected and kept confidential. The researcher audio-recorded the interviews with permission from the participants. The researcher took notes during the interviews. The final sample size was determined based on data saturation where no new information was forthcoming.

All of the interviews were transcribed. Each transcript was about 30 pages long on average and contained very rich data. After the preliminary analysis of the transcripts, the researcher invited the interviewees to confirm the transcript contents in order to assure the credibility of the data. The texts were then coded and analysed based on Colaizzi's strategy, which included reading and rereading descriptions; extracting significant statements; formulating meanings; categorizing into clusters of themes and validating with original text; describing; returning to participants; and incorporating any changes based on the informants’ feedback [37]. We adopted the Colaizzi's method as it offered a clear and systematic approach to provide a concise, yet comprehensive description of the phenomenon under study along with opportunities for participant validation [38].

This involved close reading of the interview transcripts several times and extracting significant statements and phrases which were coded, and the codes were grouped into more abstract levels of codes or themes. The analysis was done manually. The first author took the lead in coding the data, and the codes were regularly reviewed by the other authors to check for accuracy and consistency.

## Results

The views and experiences relating to foetal reduction reflected five key themes: hesitation, ambivalence and pain; the guilt of knowingly terminating a life; rituals and ceremonies to ease the sense of guilt; persuading oneself to consider the ‘big picture’; and wishing for a reunion in next pregnancy**.**

### Hesitation, ambivalence and distress

The women often regretted that they took clinical advice to implant multiple embryos for increasing their chances of pregnancy, and then having had to resort to foetal reduction. On an average, 3.75 embryos were implanted for each participant. The women’s desire to have had the right number of the implanted embryos to avoid the difficult experiences of foetal reduction was evident in the accounts:

“When receiving the implantation, the doctor told me that some may not develop into healthy embryos, therefore, he suggested to implant all of the four embryos to maximise the success rate. It was difficult to decide about the number of embryos. But I took the doctor’s advice to have a higher chance of success.” (Participant A)

“When knowing that I have had to reduce my triplets, I wish I had asked for only one implanted embryo.” (Participant B)

“I didn’t know out of the four implanted embryos, how many would be successful, hence took the doctor’s advice and had four. When I learned about the necessity of the foetal reduction, I started to think. If I had known about foetal reduction from the beginning, I might have considered not to take such risk and just asked for two embryos to be implanted.” (Participant F).

The struggles that women underwent while making decisions about foetal reduction was clearly evident in the interviews. While making the decision, there was a sense of hesitation, ambivalence and distress reflected in the views from all participants, with some women hoping that someone would tell them that they could somehow stop the foetal reduction process:

“When I found out about the foetal reduction, my mind was struggling. I wanted to see other doctors to find out if I was seriously not suitable for multifoetal pregnancy, hoping that someone could tell me the triplets could all stay.” (Participant B)

“Chorionic villus sampling showed that all of the three foetus were very healthy, so I hesitated before the reduction and wanted to keep them all. It was really hard to decide which one to lose.” (Participant C)

“I was very upset during the foetal reduction operation, and even afterwards. We made so many efforts in the test tube process and finally had the baby that we wanted, but then we had to send him off, it was really upsetting. We both hesitated over it and found it hard to lose him.” (Participant F)

Women reported instances of distress experienced by their husbands as they went through the foetal reduction procedure:

“Every embryo is my baby. It is really difficult to choose which one to give up. We had no choice but to take the foetal reduction because it was too risky for triplets. My husband cried during the operation and I asked him why, he said he was losing his own baby. (Participant A)

### The guilt of knowingly terminating a life

Women believed that they ended the fetuses’ lives knowingly and experienced strong feelings of guilt before and after the process and felt that this was against the traditional and the religious beliefs that they hold. For some women, the heartbeat represented life, and therefore, they wished to undergo the process before they recognised the signs of life:

“The traditional mind set and the religious belief taught me that the foetal reduction is wrong. I asked the doctor whether we could have the operation before the appearance of the heartbeat, if the foetal reduction is inevitable. Because heartbeat means the baby has a life already. But the doctor said the foetal reduction could only be done once the heartbeat started. That is a life.” (Participant B)

Women worried about consequences and ‘karma’, and were concerned about reincarnation of the foetal soul:

“According to Chinese tradition, foetal reduction is to terminate a life with heartbeat, so I was worried about cause and effect, and reincarnation.” (Participant C)

### Rituals and ceremonies to ease the sense of guilt

Women who experienced intense feelings of guilt relied on extensive religious ceremonies and rituals to reduce the guilt of ‘being a killer’ both before and after undertaking foetal reduction. The ceremonies and rituals included what the women described as ‘ceremony of salvation’, ‘burning incense and worship’, ‘reading Buddhist scriptures for blessing’ and ‘going to the temples to pray for safety’.

“We went to ask god before the reduction. When we decided to keep two, my husband and I had a ceremony of salvation, burning the incense and worship, to keep our mind in peace.”(Participant C)

“I read Buddhist scripts for those two reduced foetuses. In fact, I started the reading before the operation and continued afterwards, for peace of mind. I don’t know if reincarnation exists or not. Even though they were not born, they did have heart beat so I hope they could go to a nice place.” (Participant B)

“I believe in Buddhism. I was reading scripts to pray for a smooth pregnancy and to ask blessing for the reduced fetus, because they could not come to this world together. I prayed for blessing through reading the scripts and even went to the temples to pray for safety, but I didn’t do any ceremony at the temples for the baby spirit.” (Participant F)

“At the Chungyuan Festival, there will be a Purdue ceremony. I went to the temple and set up a card for my child, praying and hoping that he will be taken care of by the gods since the reduction.” (Participant G)

### Persuading oneself to consider the ‘big picture’

With their feelings of guilt, women often tried to persuade themselves to look at the ‘big picture’ in terms of the overall outcome and to consider foetal reduction as something they should not have normally done, but have had to do to maximise the overall benefit:

“I had to tell myself that was necessary. Without the reduction, the remaining twins would be left in risk, so it was to benefit the twins. I adjusted my mood and then had the foetal reduction done.” (Participant A)

“It is important that you have something in mind to support yourself. I had to consider the remaining two hence kept telling myself that the sacrifice was to protect the other two babies.” (Participant B)

“Reduction is for the benefits of the other two. After all, reduction had to happen so I didn’t think too much, treating it as a set route. The foetal reduction cost a baby’s life but without reduction, it will cost three.” (Participant E)

Participants also reflected on how they tried to gear their thoughts back to focus on the health and safety of the remaining babies:

“My husband and I finally adjusted our thoughts and agreed that the reduction is great love to the remaining babies.” (Participant D)

“It was impossible to look after those reduced two. I understood that decision was necessary. I was praying for them in my mind and decided to look after the remaining two babies. I felt I had to adjust my mood quickly. After all, it was risky to take that operation and if anything happened to the live babies, I would have even bigger problems. So I had to think for the remaining one.” (Participant F)

### Wishing for a reunion in the next pregnancy

The experience of foetal reduction, and the loss of their unborn babies, was expressed as an unforgettable incident that was hard for the women to get over. Women mentioned about themselves and their partners thinking about the lost foetuses for a long time following the procedure and wishing for a reunion with the lost foetuses in their next pregnancy:

“My husband and I still thought about it after a period of time. Sometimes my husband prays for the baby to go to the heaven and find another opportunity to come back to us. Now that our twins are doing quite well, we couldn’t help but thinking that we might just be the same to keep three of them. We could never forget about the lost baby girl (in tears)” (Participant D)

“Up to now when I think of him, I imagined he maybe look like me or my husband or looks like a combination of us. I don’t know who he is like more” (Participant G)

## Discussion

This paper builds on the evidence on first-time mothers’ experiences of conception and motherhood following ART treatment in Taiwan and examines the in-depth experiences of women who underwent foetal reduction. As this was a qualitative study based on the experiences of seven participants, our findings may not necessarily be representative of the experiences of all women who have undergone foetal reduction in Taiwan or elsewhere. However, our methodological approach created a suitable “space” for these women to freely express their experiences.

Five key themes emerged in the study with respect to the experiences of undergoing foetal reduction: feelings of hesitation, ambivalence and distress; the guilt of knowingly terminating a life; rituals and ceremonies to ease the sense of guilt; persuading oneself to consider the ‘big picture’; and wishing for a reunion in next pregnancy.

Despite the increased chances of maternal and foetal complications of twin and higher order multiple births [2, 4–7], and existing regulatory restrictions of a maximum embryo transfer [39–41], Embryo implantations performed during the blastocyst stage typically involve fewer embryos and thus reduce the risk of multiple pregnancy [42, 43]. Single embryo transfer is increasingly being used in assisted reproduction. However, embryo quality and advanced maternal age are factors that affect the embryo-transfer decisions of attending physicians. In countries such as Taiwan where assisted reproductive treatments (ART) are not normally covered by standard insurance policies, medical teams are particularly sensitive to the efficacy of ART outcomes, especially because not all embryos are expected to reach the blastocyst stage in vitro. Non-blastocyst transfers account for 85.71% of the embryo transfers addressed in this study, with embryo quality and procedural loss found to be the principle factors limiting implantation success. Achieving comprehensive implementation of the single-embryo transfer approach is difficult in countries that do not provide insurance coverage for ART. The subsequent foetal reduction poses severe ethical dilemmas for couples as evidenced in our study. Researchers have highlighted the tensions, uncertainties and challenges that providers and parents face while making decisions about the number of embryos to transfer [44], and it has been suggested that providers may choose to transfer more embryos following patient requests [45]. The decisions concerning the number of embryos to transfer during ART treatment was not directly explored in our study. However, the accounts from the participants suggest the need for more detailed discussions between providers and the women regarding the number of embryos to transfer and the potential likelihood of foetal reduction in cases of multiple embryo transfer.

The heightened sense of vulnerability that the mothers in our study experienced during the foetal reduction process and their feelings of intense guilt could inevitably cause huge stresses on women’s psychological health. It has been reported in previous studies that women who conceived using ART tend to experience higher levels of anxiety compared to those who conceived naturally [46–49]. While the safety and health of the foetus was a primary concern for the women in our study, the feelings of hesitation, ambivalence, distress and the guilt of knowingly terminating a life that the women underwent both before and after the process of foetal reduction could all be the starting point for interventions for these women. The observance of rituals and ceremonies that the women resorted to as a major coping method to ease the sense of guilt, and their notions of an expected reunion in the next pregnancy bring into fore the importance of understanding and taking into account the broader cultural and religious context while planning interventions to support these mothers. The Confucian ideals of filial responsibility to continue the family line, Buddhist beliefs in the sinfulness of killing life, and dominant Taiwanese cultural ideals of women as nurturers, make the decision to abort a foetus filled with guilt and regret [50]. Researchers have noted that women who experienced induced abortion often worshiped the aborted lives to ease the guilt and the pain by chanting and making confessions. Many of these rituals are conducted by both Buddhist and Taoist institutions with variations among regions, and places of worship such as temples and shrines [51]. Reciting scripture or worshiping the ceremonial tablet was seen to be an expression of women’s repentance. Women believed that they helped the dead foetuses to get ready for the reincarnation through religious activities [50–52].

The impact of Taiwanese society on pregnancy following ART has been indicated by a previous study [49]. Persuading oneself to consider the ‘big picture’ coupled with views of foetal reduction as a possible measure to ensure the health of their foetus is a key point for health care professionals to initiate positive thinking among these women and to reduce the resultant psychological distress from the process. The findings from the bigger study showed that the participants viewed the pregnancy as a family event, and the intense practical and emotional support from the extended family was a major resource that helped the women cope with the demands of the ART treatment and the pregnancy [36].

This is one of the very few qualitative studies that have explored the experiences of women who underwent foetal reduction following ART treatment in Taiwan. The qualitative approach offered the opportunity for an in-depth exploration of the experiences, but the findings may not be considered generalisable due to the nature of the study and the limited sample size obtained through purposive sampling method. The likely influence of the interviewer on the research process is another aspect that needs to be considered in qualitative research [53] as the relationship may encourage or inhibit the interviewee to share their personal and intimate experiences with the interviewer. The first author (MZH) who conducted the interviews worked as a consultant at the infertility unit where the women were recruited. While this helped the interviewer to build a trusting relationship with the interviewees and facilitated the recruitment and the interviews, this could potentially introduce some courtesy bias into the findings. In order to enhance the rigour, we purposively included the most appropriate participants for this aspect of the study, and the chosen study method enabled collection of rich data that was relevant and useful in achieving the research objective. Care was also taken to ensure that sufficient good quality data were collected prior to concluding that data saturation was achieved [54]. In order to enhance the reliability of the results, the researcher invited the participants to validate the accuracy of the preliminary findings. Regular discussions within the team that included experts in the subject areas of obstetric nursing, infertility, public health and maternity care as well as qualitative methodological approaches also enhanced the scientific rigour of the research process.

## Conclusion

The key themes reflected in the women’s accounts provide an in-depth understanding of first-time mothers’ experiences of undergoing the foetal reduction procedure following ART treatment and provide unique insights into their thoughts and feelings that have not been addressed by previous studies. The findings offer useful insights for policy, practise and future research in this area. Multi-foetal gestations remain one of the key contributors of adverse perinatal outcomes after ART and as little can be done after multiple embryo transfer, there is an explicit need for efforts at different levels including appropriate legislative measures supplemented by provider and patient awareness to limit the number of embryos that can be transferred during ART. ART providers should undertake a more sensitive approach that involves detailed discussions with women and their families along with a precise recognition of risks to tailor the embryo transfer process to suit individual women’s needs.

Women who undergo foetal reduction should be provided with individually tailored interventions taking into account the cultural and religious context towards enhancing their coping strategies before and after foetal reduction. This should include support from, and access to trained professionals such as registered nurses and psychologists in addition to ART clinicians. Future studies should examine perspectives of patients and clinicians with respect to multiple embryo transfer and the nature of interventions both to limit multiple embryo transfer and to reduce the physical and psychological distress for women and their families who undertake foetal reduction. The perspectives of fathers and the extended family members in making decisions about the number of embryos transferred and the foetal reduction process are worth exploration.

## Data Availability

The transcripts contain confidential information and therefore are not available to share.

## Abbreviations

ART: Assisted reproductive technology
IVF: In vitro fertilisation
